# Supplementary material for: MetaQTL: a package of new computational methods for the meta-analysis of QTL mapping experiments
Source: BMC Bioinformatics. 2007 Feb 8;8:49. doi: 10.1186/1471-2105-8-49 (PMC1808479; doi:10.1186/1471-2105-8-49)
Supplement: Additional File 7 — MetaQTL Package : jar file and tutorial. This Zip archive contains both the MetaQTL JAR file and the files of the tutorial. [file 1471-2105-8-49-S7.zip › org.inra.metaqtl/doc/org/thalia/bio/util/package-summary.html]

org.thalia.bio.util


|  |  |  |
| --- | --- | --- |
| **Overview** \| **Package** \| Class \| **Use** \| **Tree** \| **Deprecated** \| **Index** \| **Help** | |  |
| **PREV PACKAGE**   NEXT PACKAGE | **FRAMES**    **NO FRAMES**     **All Classes** |  |


---

## Package org.thalia.bio.util

| **Interface Summary** | |
| --- | --- |
| **ILGroupPosition** | Class Description Here |

| **Class Summary** | |
| --- | --- |
| **BioCrossTypes** |  |
| **BioEntityNameComparator** | Class Description Here |
| **BioLocusTypes** |  |
| **BioUtilities** | General comments must be added here. |
| **EstimatedLocusPosition** | Class Description Here |
| **LGroupPosition** | Class Description Here |
| **LocusPositionComparator** | Class Description Here |
| **NumberFormat** |  |
| **StaticLocusPosition** | Class Description Here |
| **TabulatedReader** | This class can be used to parse tabulated file in which the first line contains the label of the tabulated fields. |
| **TabulatedWriter** | General comments must be added here. |

---


|  |  |  |
| --- | --- | --- |
| **Overview** \| **Package** \| Class \| **Use** \| **Tree** \| **Deprecated** \| **Index** \| **Help** | |  |
| **PREV PACKAGE**   NEXT PACKAGE | **FRAMES**    **NO FRAMES**     **All Classes** |  |


---
